# Supplementary figures and images for: Rat Hepatocytes Mitigate Cadmium Toxicity by Forming Annular Gap Junctions and Degrading Them via Endosome–Lysosome Pathway
Source: Int J Mol Sci. 2022 Dec 9;23(24):15607. doi: 10.3390/ijms232415607 (PMC9778680; doi:10.3390/ijms232415607)

Supplementary Materials S1: Optimal TPA Concentration Screening by CCK8 :

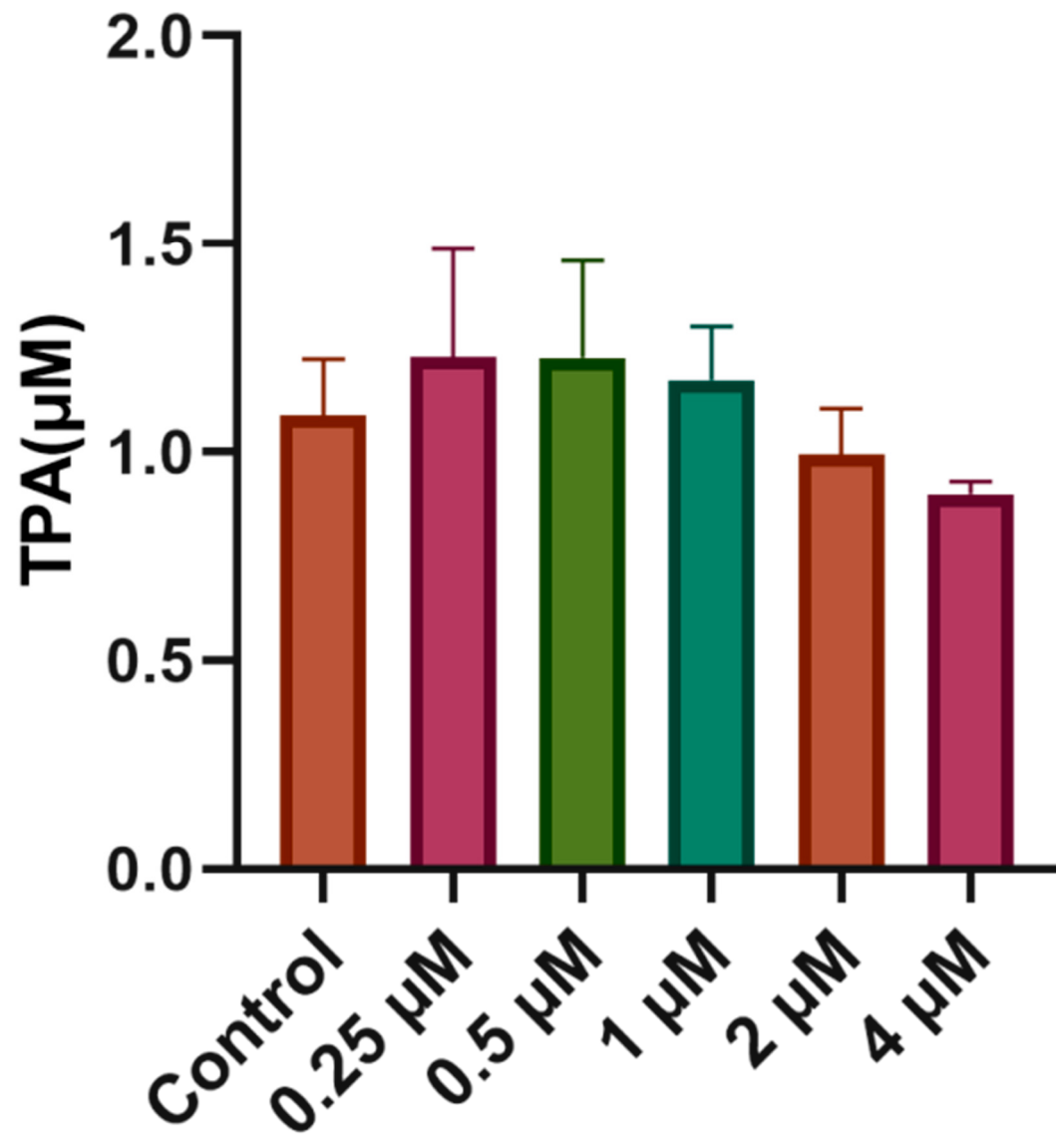

Supplement: Supplementary file 1 [file ijms-23-15607-s001.zip › ijms-2058599-supplementary.pdf]
